# Use of Low-Cost Spherical Cameras for the Digitisation of Cultural Heritage Structures into 3D Point Clouds

**DOI:** 10.3390/jimaging8010013

**Published:** 2022-01-17

**Authors:** Sorin Herban, Domenica Costantino, Vincenzo Saverio Alfio, Massimiliano Pepe

**Affiliations:** 1 Poliltehnic University of Timisoara, Traian Lalescu 2°, 300223 Timisoara, Romania; sorin.herban@upt.ro; 2 Dipartimento di Ingegneria Civile, Ambientale, del Territorio, Edile e di Chimica, Polytechnic of Bari, Via E. Orabona 4, 70125 Bari, Italy; domenica.costantino@poliba.it (D.C.); vincenzosaverio.alfio@poliba.it (V.S.A.)

**Keywords:** spherical photogrammetry, equirectangular, Ricoh Theta, point cloud, TLS, virtual tour

## Abstract

The digitization of Cultural Heritage is an important activity for the protection, management, and conservation of structures of particular historical and architectural interest. In this context, the use of low-cost sensors, especially in the photogrammetric field, represents a major research challenge. In this paper, the use of cameras capable of capturing a 360° scene with a single image was assessed. By using spherical photogrammetry and the algorithm based on the structure from motion and multi-view stereo, it is possible to reconstruct the geometry (point cloud) of an object or structure. In particular, for this experiment, the Ricoh theta SC2 camera was used. The analysis was conducted on two sites: one in the laboratory and another directly in the field for the digitization of a large structure (Colonada in Buziaș, Romania). In the case study of the laboratory, several tests were carried out to identify the best strategy for reconstructing the 3D model of the observed environment. In this environment, the approach that provided the best result in terms of both detail and dimensional accuracy was subsequently applied to the case study of Colonada in Buziaș. In this latter case study, a comparison of the point cloud generated by this low-cost sensor and one performed by a high-performance Terrestrial Laser Scanner (TLS), showed a difference of 15 centimeters for 80% of the points. In addition, the 3D point cloud obtained from 360° images is rather noisy and unable to construct complex geometries with small dimensions. However, the photogrammetric dataset can be used for the reconstruction of a virtual tour for the documentation and dissemination of Cultural Heritage.

## 1. Introduction

The use of low-cost sensors for photogrammetric purposes is an important research topic since it is used in multiple application fields, such as robotics, mobile mapping, digitization of Cultural Heritage (CH), etc. [1,2,3,4,5]. In recent years, omnidirectional cameras, i.e., cameras with a 360° field of view (FOV) in the horizontal plane, are becoming more and more widespread. Depending on the construction of the omnidirectional camera, it is possible to obtain 3 types of sensors: dioptric, which uses a combination of shaped lenses (e.g., fisheye lenses); catadioptric, which combines a standard camera with a shaped (parabolic, hyperbolic, or elliptical) mirror; and polydioptric, which uses multiple cameras with overlapping FOV [6]. In the present paper, a polydioptric system was used, and specifically a Ricoh Theta camera, which has two fisheye lenses [7]. In the images generated by these cameras, feature distortion increases non-linearly from the center to the side of the images and equirectangular projection is generally used to represent the captured scene. In the field of photogrammetry, a key role in the construction of an accurate and precise 3D model is played by camera calibration. Many methods, toolboxes, and libraries have been proposed to estimate Internal Orientation Parameters (IOPs), varying according to the image acquisition configuration, the mathematical model (such as standard, extended and generic), the statistical approach, and the level of automation. However, simultaneous camera calibration for IOP and Relative Orientation Parameters (ROPs) estimation has not been taken into account in existing MATLAB toolboxes for fisheye lens calibration or even in commercial software [8].

Argyriou et al., 2017 [9] wrote about how a novel geometric calibration of the full spherical image is presented; IOPs are defined for defined network points and subsequently added to the measured control points in image space as correction terms to be used in collinearity equations, in the form of spherical coordinate systems, to obtain direction vectors between image points and object points. Campos et al., 2018 [8] presented a technique for the calibration of a 360° dual imaging system (Ricoh Theta S) which is based on calibration with a bundle adjustment and using the equidistant fisheye lens model and relative orientation stability constraints, such as distance, base elements and rotation matrix between individual cameras’ coordinate systems. Campos et al., 2019 [10] proposed an approach to boost feature-based matching techniques on fisheye images with recursive reduction of the search space based on epipolar geometry; in this latter paper, the Ricoh Theta S was used for several tests. Liu et al., 2020 [11], in order to improve the computational efficiency and reliability of the correspondence, propose a spherical 3D panoramic epipolar line based on the essential matrix. This method can be divided into three main steps, i.e., first, the essential matrix of computer vision principles is used to establish the epipolar geometry model of the spherical panoramic image; then the mathematical equations of the epipolar line are derived and finally, the epipolar image distribution law statistics are calculated.

This paper intends to continue with the line of research based on camera calibration and, more in general, aims at identifying photogrammetric techniques that allow 3D models to be obtained from spherical images and on the combination based on Structure from Motion (SfM) and Multi-View Stereo (MVS) algorithms. SfM solves the issue of camera positioning and scene geometry simultaneously and automatically, using a highly redundant bundle adjustment based on matching features in multiple overlapping, while MVS algorithms allow densifying the point cloud generated by the SfM approach [12]. Until a few years ago, while SfM has been studied extensively for perspective cameras and omnidirectional hemispheric cameras, little attention has been given to fully spherical cameras [13]. Nowadays, most SfM photogrammetric software such as Agisoft Metashape, Pix4D, Reality Capture, etc. integrates either spherical or panoramic images [2,14,15]. For this reason, this study aims to apply SfM-MVS algorithms to build a 3D model with special regard to applications in the field of CH. Indeed, the digitization of CH through the use of low-cost sensors and software based on the SfM-MVS approach is growing. Previous and recent applications in the CH field can be found in several papers [16,17,18,19,20]; however, the results do not show unambiguity on the geometric quality of the point cloud. In fact, if on the one hand, they show the ease of use of the panoramic camera and the ability of the system to produce photogrammetric models, on the other hand, the geometric accuracy of the 3D reconstruction shows some limitations. Therefore, the aim of the paper is to investigate the potential of spherical photogrammetry using a low-cost camera in 3D reconstruction and documentation of CH sites.

## 2. Equirectangular Projection

**Figure 1:**
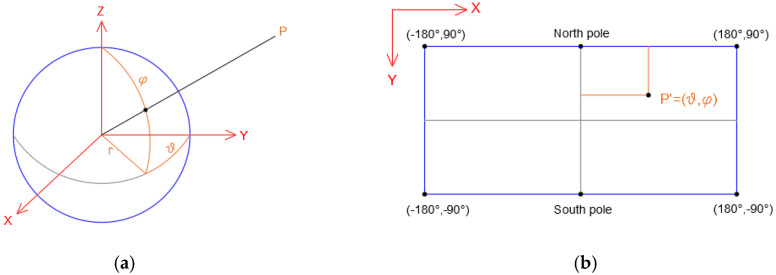
Relationship between spherical coordinates (**a**) and the image coordinate in the equirectangular image (**b**).

The equirectangular projection (also called the *equidistant cylindrical* projection, *geographic* projection, or *la carte parallélogrammatique* projection) was born in the world of cartography [21]. Indeed, this map projection is attributed to Marinus of Tyre, who Ptolemy claims invented the projection around AD 100 [22]. This cylindrical projection converts the globe into a Cartesian grid where each rectangular grid cell has the same size, shape, and area. In this case, it uses the equator (Plate Carrée projection) as the central parallel and the grid cells are squares. Therefore, in this projection, the meridians are set as equally spaced vertical lines while the parallels are differently spaced horizontal lines. Another important feature of this projection is that all the graticule intersections are 90°, i.e., all the lines of the graticule are perpendicular to each other (Figure 1a). The scale is true along the equator or along the two parallels equidistant from the equator. In addition, the distortion increases as the distance from the standard parallels increases. In the field of cartography, this projection is used for simple portrayals of the world or regions with minimal geographic data or even for city maps or other small areas with large map scales. In the last few years, thanks to the development and progress in the field of photogrammetry, remote sensing, and computer vision, the equirectangular projection has been applied to many fields, such as the Digital Cultural Heritage (DCH) [23]. In this field, a notable contribution was provided by the spreading of spherical photogrammetry, which allowed obtaining a 360° view of the surrounding environment. Indeed, by taking more photographs (partially overlapping) to 360° from a single point of view, it is possible to obtain a rendered panorama where the images are projected onto a virtual sphere, which, in turn, can be projected onto a plane. In this way, it is possible to obtain the so-called equirectangular projection [24]. Since this type of projection originates in the cartographic world, it follows that even the nomenclature to describe the passage from the sphere to the plane uses merely cartographic terms, such as latitude and longitude. From a mathematical point of view, considering a sphere with a radius *r*, spherical coordinates latitude φ and longitude ϑ, this can be projected on the cartographic plane map of coordinates x, y using following the relations:

(1) $$\begin{array}{c} x=r\cdot \vartheta \\ y=r\cdot \varphi \end{array}$$

Therefore, the height of the map is equal to the development of a meridian (πr) while the base is equal to the circumference of the sphere (2πr), as shown below (Figure 1b).

Given the Cartesian coordinates of a point P(X,Y,Z), a Cartesian terrestrial reference system (whose axes are $X_{0},\;Y_{0},Z_{0}$) and another Cartesian reference system centered in the sphere and parallel to the other reference system taken into consideration (whose axes are $X^{\prime }$,$\;Y^{\prime }$,$\;Z^{\prime }$) it is possible to obtain the relations:

(2) $$\begin{array}{c} \;X\prime =X-X_{0}\\ \;Y\prime =Y-Y_{0}\\ \;Z\prime =Z-Z_{0} \end{array}$$

In addition, considering another Cartesian reference system centered in the sphere (whose axes are $X^{*},\;Y^{*},\;Z^{*}$,) but inclined by a certain angle, it is possible to obtain the following relations [25]:

(3) $$\begin{array}{c} X^{*}=d\cdot \sin\varphi \cdot \sin\theta \\ Y^{*}=d\cdot \sin\varphi \cdot \cos\theta \\ Z^{*}=d\cdot \cos\varphi \end{array}$$

where d is the distance of the sphere center O from point P, invariant in the two reference systems. The distance d can be calculated by the Pythagorean Theorem:

(4) $$d=\sqrt{{\;X\prime }^{2}+{\;Y\prime }^{2}+{\;Z\prime }^{2}}=\sqrt{X^{*}{}^{2}+Y^{*}{}^{2}+Z^{*}{}^{2}}$$

The coordinates of the point *P* in the $X^{*},\;Y^{*},\;Z^{*}$ the system can be obtained as follows:

(5) $$\left[\begin{array}{c} X^{*}\\ Y^{*}\\ Z^{*} \end{array}\right]=\left[\begin{array}{ccc} 1 & {\mathrm{da}}_{z} & {\mathrm{da}}_{y}\\ -{\mathrm{da}}_{z} & 1 & {\mathrm{da}}_{x}\\ {\mathrm{da}}_{y} & -{\mathrm{da}}_{x} & 1 \end{array}\right]\left[\begin{array}{c} X-X_{0}\\ Y-Y_{0}\\ Z-Z_{0} \end{array}\right]=\left[\begin{array}{c} d\cdot \sin\varphi \cdot \sin\theta \\ d\cdot \sin\varphi \cdot \cos\theta \\ d\cdot \cos\varphi \end{array}\right]$$

where ${\mathrm{da}}_{x},\;{\mathrm{da}}_{y},{\mathrm{da}}_{z}$ represent the correction angles. 

The equation 5 can be written in another way, as shown below:

(6) $$\left[\begin{array}{c} X^{*}\\ Y^{*}\\ Z^{*} \end{array}\right]=\left[\begin{array}{ccc} r_{1} & r_{2} & r_{3}\\ r_{4} & r_{5} & r_{6}\\ r_{7} & r_{8} & r_{9} \end{array}\right]\left[\begin{array}{c} X-X_{0}\\ Y-Y_{0}\\ Z-Z_{0} \end{array}\right]=\left[\begin{array}{c} d\cdot \sin\varphi \cdot \sin\theta \\ d\cdot \sin\varphi \cdot \cos\theta \\ d\cdot \cos\varphi \end{array}\right]$$

From the previous relation (see Equation (6)), dividing the equation $X^{*}$ by $Y^{*}$, we obtain:

(7) $$\vartheta =\mathrm{atg}\frac{r_{1}\left(X-X_{0}\right)+r_{4}\left(Y-Y_{0}\right)+r_{7}\left(Z-Z_{0}\right)}{r_{2}\left(X-X_{0}\right)+r_{5}\left(Y-Y_{0}\right)+r_{8}\left(Z-Z_{0}\right)}$$

Instead, dividing the equation $Z^{*}$ by d (from the relation 6), it is possible to obtain:

(8) $$\varphi =\mathrm{acos}\frac{r_{3}\left(X-X_{0}\right)+r_{6}\left(Y-Y_{0}\right)+r_{9}\left(Z-Z_{0}\right)}{d}$$

Relations (7) and (8) are the equations of collinearity for the spherical panorama [26].

## 3. Materials and Methods

### 3.1. 360° Camera Used for the Experimentation: Ricoh Theta SC2

At the moment, new 360° cameras are available on the commercial market, able to capture the whole surrounding environment in one shot. In the following Table 1 are listed some 360° cameras according to the resolution and the relative cost.

Therefore, the choice of camera derived from a compromise between technical performance and price. Ricoh Theta SC2 (Ricoh Company, Ota, Tokyo), used in the experiment, met the desirability of price, resolution, focal length, etc. 

Ricoh Theta SC2 is a mid-range 360° camera and consists of two cameras, with one f/2 lens facing forward and another facing backward. The main technical features of the Ricoh Theta SC2 are shown in Table 2. 

**Figure 2:**
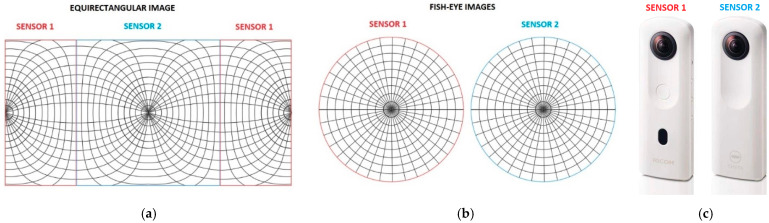
Geometric configuration of the equirectangular image (**a**) using fisheye lenses (**b**) and generated by the Ricoh Theta SC2 (**c**).

The two sensors with fisheye lenses allow building the panoramic image. In particular, the part covered by the individual sensors can be schematized as follows (Figure 2).

### 3.2. Pipeline of the Investigation Method

The 3D reconstruction of an object can be performed taking into account the calibration of the fisheye lens or using the collinearity equation for spherical images. To adjust the lens, it is necessary to perform a calibration using a checkerboard or an appropriate test site. In general, the calibration of a camera used for photogrammetric purposes should be performed periodically especially for low-cost cameras where the instability in the internal orientation suggests the repetition of the calibration every time a new photogrammetric survey is about to be performed. An optimal solution would be that the calibration test images should be acquired at the same time as the survey, avoiding the need to turn off the camera and change its optical attitude. Therefore, the calibration process is important for the success of photogrammetric surveying. However, considering this type of camera, the first step is to decompose the equirectangular image into the original image, i.e., into 2 fisheye images. This is achieved by building an algorithm capable of dividing the image generated by the Ricoh Theta SC2 camera. If it is not possible to obtain satisfactory results from the generation of fisheye images and the calibration process, it is necessary to take into consideration spherical photogrammetry. In this way, it is possible to estimate the photogrammetric parameters directly on the job or even better during the 3D processing using spherical images. This means obtaining a 3D reconstruction of an object simply and automatically by using dedicated software (although there are not many programs of this type commercially available at the time of writing). For example, Agisoft Metashape (Version 1.5.1) is a professional 3D processing software, supporting panorama as an input source, able to build a 3D model using the collinearity for the spherical panorama (see Equations (7) and (8)). In the Agisoft Metashape software, the automatic processing steps that lead to the construction of the 3D model are the alignment of the images and the building of a dense Point Cloud. In this environment, the creation of masks on the lower part of the images and the sky when taken outdoors can be a good strategy to increase accuracy and decrease the total alignment time. To better analyze the behavior of the SfM-MVS algorithms using spherical images generated by the Ricoh Theta SC2 camera, the test is performed in two different sites, firstly carrying out an initial test in the laboratory and then on a structural site belonging to the CH environment. On the laboratory site, it is possible to analyze the impact of the choice of calibration type on the quality of the 3D model. Subsequently, in consideration of the results obtained, field experimentation can be performed. In particular, it was decided to analyze the quality of the photogrammetric point cloud generated by the Ricoh Theta in comparison to the point cloud generated by Terrestrial Laser Scanner (TLS). In this way, it is possible to evaluate the difference between the point clouds and, more in general, to identify the level of quality of the photogrammetric process using a low-cost sensor. The CH site used for the test is located in Buziaș (Romania).

#### 3.2.1. Laboratory Test (Indoor Environment)

The first test was conducted in a room (geometrically simple and regular) of the geomatics laboratory of the Polytechnic of Bari (Italy) where 20 targets were placed on interior walls, floor, and ceiling. The coordinates of the target were calculated through a post-processing operation carried out in Leica Cyclone on one scan (with a maximum distance of 5m) performed using the HDS3000 Terrestrial Laser Scanner which has a position accuracy of 6mm@50m [27]. Subsequently, 9 spherical photos performed by Ricoh Theta SC2 were taken inside the room.

When using the self-calibration approach, it is necessary to calibrate the optics of the camera. In particular, the calibration of the 9 images produced by the camera was carried out in 2 steps; firstly, the equirectangular image was divided into two images and then the individual images were transformed into fisheye images. This task was carried out by writing code in Python language (Version 3.8). The algorithm developed is intended as a Python Script to convert equirectangular images to double fisheye images using OpenCL (Version 3.0) for bulk processing, and can be used with any hardware configuration capable of running the OpenCL runtime. This script is inspired by Paul Bourke’s work on dual fisheye conversion, implementing an inverse procedure from his method [28]. In line with Bourke’s work, the algorithm will perform a pixel-wise transformation from the equirectangular/spherical representation of the 360° images to the double fisheye one. The script will split the original images into two hemispheres, then each pixel position is normalized to a 2D vector space, and based on each pixel position on the fisheye image, we can easily calculate the corresponding pixel on the equirectangular hemisphere. This was originally implemented as an iterative method using the NumPy package (Version 1.21.0). Moreover, a more efficient solution can be found using OpenCL to compute each pixel on a different thread of a GPGPU capable device. The script requires the following packages to run: Pillow, PyOpenCL, and NumPy. It may also require the installation of the Developer Drivers for OpenCL from the official CUDA, AMD or Intel websites in order to correctly compile the OpenCL code for the specific hardware used. The code is reported in Appendix A. Fisheye and equirectangular images were imported and processed in the Agisoft Photoscan software. The software was unable to align a few images, but even those that appear to be aligned are actually positioned incorrectly. In order to increase the probability of success of the fisheye in the bundle adjustment process, an additional 4 spherical images were added to the project (8 fisheye images). However, the photogrammetric processing did not lead to a suitable result for the reconstruction of a 3D model. 

Using 360° images, instead, the software is able to correctly align spherical images. Regarding the accuracy achievable by the camera (evaluated on the targets), a Root Mean Square Error (RMSE) value of about 3 centimeters was obtained. In particular, RMSE values for all the cameras are summarized in the following Table 3.

**Figure 3:**
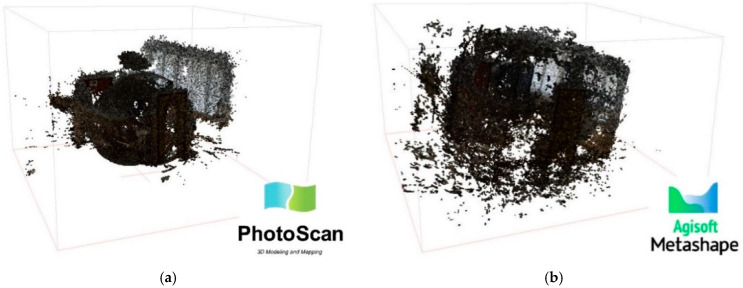
Results of point cloud (room of the laboratory) processing in Agisoft Photoscan (**a**) and Agisoft Metashape (**b**).

After the images were aligned, Agisoft Photoscan (Version 1.4.4) generates, according to the Multi-View Stereo (MVS) approach, a dense cloud. The dense point cloud generated in this software is based on depth maps [29] and calculated using dense stereo matching. Depth map filtering in Metashape evaluates pairwise depth maps for matched images using a connected component filter, which analyzes segmented depth maps based on the distance of a pixel from the camera [30]. Combined depth maps generated for each camera are transformed into partial dense point clouds, which are then merged into a final dense point cloud with additional noise filtering steps applied in the overlapping regions. The point cloud generated in the Photoscan environment was affected by noise and by an inaccurate geometric reconstruction of the environment detected (see Figure 3a). In order to obtain a wider assessment of the results, another processing was carried out, with the same settings and the same GCPs and CPs, but using Agisoft Metashape PRO. In this updated version of the software, the filtering algorithms have been completely updated, guaranteeing a significant reduction in noise and preserving the finest details of the surfaces. Indeed, new algorithms developed in Metashape contribute to a significant reduction in noise, while preserving the finest surface details. Metashape takes full advantage of Graphics Processing Unit (GPU) acceleration, substantially reducing processing time and memory consumption. In addition, Metashape is optimized for multi-core Central Processing Units (CPUs) and multiple GPU systems for fast generation of results using numerous images. The result was still a noisy point cloud, but compared to the one processed with the previous version, it reconstructed more accurately the geometry and a few details of the survey object (Figure 3b).

In terms of accuracy on GCPs and CPs, both software generated the same order of magnitude, while a greater difference was found on Tie Point and Dense Cloud density. Table 4 below summarises the main values obtained from the comparison.

The two models were then compared, following a Cloud to Cloud (C2C) approach using Cloud Compare software. With this procedure, it was possible to calculate the distance between the two clouds, setting as reference cloud the one obtained from Agisoft Metashape software, and as comparison cloud the one obtained from Agisoft Photoscan. With this procedure, the approximate distances useful for defining the best octree level are calculated first, and then the real distances are calculated. The accuracy evaluated on GCPs was a few centimeters. However, the photogrammetric model was not able to describe the geometry of the entire room; better results were obtained using Metashape instead of Photoscan. Indeed, Agisoft Metashape was more accurate and defined also in the reconstruction of the floor and ceiling and a few elements inside the room, confirming the greater efficiency of its algorithms. In addition, in order to verify the incidence of the number of images in the reconstruction of the surrounding environment, a new dataset of photos was acquired. Additional photos were acquired at the same (planimetric) position but at a higher height, i.e., more images were taken from a tripod. 5 datasets with different spatial configurations (A, B, C, D, E) were created, as shown in Table 5. In particular, for each dataset, it was analyzed the number of tie points and the dense cloud as the number of images varied.

**Figure 4:**
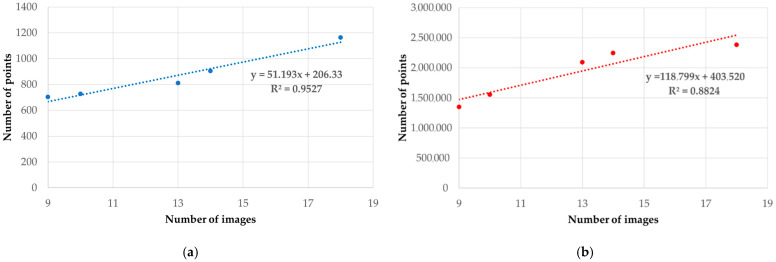
Influence of the number of images in the photogrammetric process versus tie point (**a**) and versus dense cloud (**b**).

From Table 5, it can be seen that from a configuration of 9 cameras at ground level, the number of tie points and dense cloud increases with the number of images. For example, in dataset E, adding 9 images to the initial configuration (dataset A) we obtain a percentage increase equal to 66% of the tie points and 77% of the dense cloud. The change in the number of tie points and the dense cloud in relation to the increase in the number of images is shown in Figure 4. In addition, a possible functional relationship between the number of images and tie points (Figure 4a) and a number of images and dense cloud (Figure 4b) was evaluated by fitting linear regression. From the analysis of these graphs, it is possible to notice a strong correlation between the variables taken into consideration; tie point and dense cloud with respect to the number of images show R^2^ values of 0.95 and 0.88 respectively.

**Figure 5:**
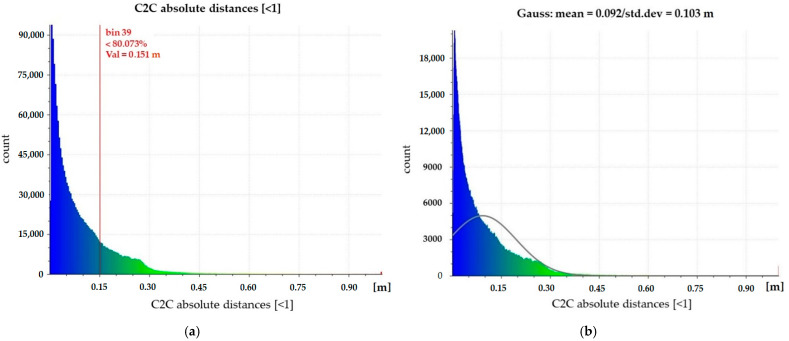
C2C comparison between photogrammetric and TLS data: C2C (**a**) and Gauss distribution (**b**).

Therefore, the contribution of the new dataset of images was important in defining the geometry of the room. By comparing the dense point cloud with the one generated with only the images taken at a low height, an improved definition of the surfaces was obtained; however, even in this case, the point cloud was noisy. To evaluate the quality of the point cloud generated by the photogrammetric process using 18 images, a C2C was performed between the dataset with the spherical images and the TLS dataset (Figure 5).

This comparison showed that about 80% of the points have a distance of less than 15 cm (Figure 5a). Furthermore, the Gaussian distribution of the points shows a mean value of 0.092 m and a standard deviation value of 0.103 m (Figure 5b).

In order to verify the performance of this system in a more complex and larger environment, a CH site in Romania was chosen for testing. Taking into account the results obtained in the laboratory test, the Agisoft Metashape software, two levels of height for the acquisition of the images, and the spherical photogrammetry approach were used for further processing.

#### 3.2.2. Cultural Heritage Datasets

The site used for the experimentation was the Colonnade in Buziaș, Timiș County (Romania).

The Colonnade was built in 1875 by order of Emperor Franz Joseph to make Empress Sissi’s walks in the park more pleasant. The Buziaș Colonnade was built with wood in the Byzantine style and encircles the resort park, measuring 500 meters in total. In recent years, the Buziaș Colonnade had fallen into disrepair and was in danger of destruction until 2015, when restoration work began, which lasted about 2 years and restored the structure to its former splendor.

In order to assess the quality of the point cloud obtained by the Ricoh Theta camera, a comparison was performed with the point cloud generated by TLS, which acquires spatial coordinates of numerous points on an object by emitting laser pulses toward these points and measuring the distance from the device to the target [31]. The TLS used for the experimentation was the Z+F IMAGER^®^ 5010C manufactured by Zoller and Fröhlich GmbH, Wangen, Baden-Wurttemberg, Germany. The 5010C is a phased system using a class 1 infrared laser. Compared to other TLSs, the 5010C has an exceptionally high and fast data acquisition rate of 1.06 million points per second while maintaining a linearity error of less than 1 mm (within 20 m of the surface). In addition, TLS has an approximate range of 187 meters and is able to acquire point data with a vertical FOV of 320° and a horizontal FOV of 360°. To cover the entire structure, 42 scans were carried out. The post-processing was performed in the Z+F LaserControl software (rel. 9 Office version); the scans were aligned manually using as reference points the flat targets that had been accurately positioned inside the investigated site.

The alignment phase of the scans resulted in a total of about 280 million points. The point cloud was georeferenced in the UTM34T-WGS84 (EPSG: 32634) cartographic system using GCPs surveyed using GNSS (Global Navigation Satellite Systems) technology. In order to easily manage the point cloud, 4 files in .las format were created.

**Figure 6:**
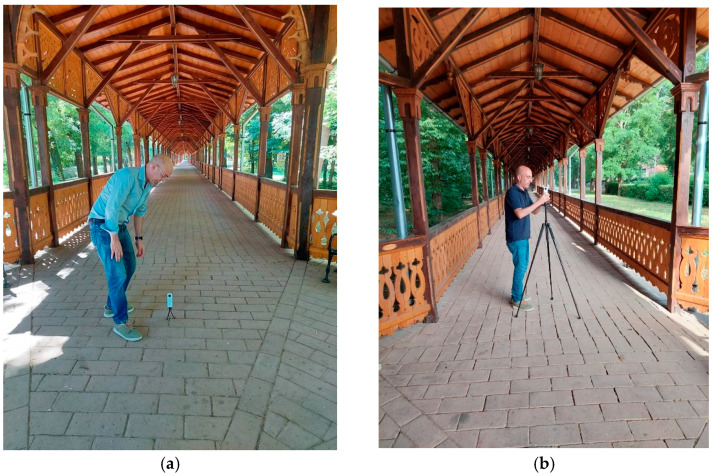
Survey by Ricoh Theta SC2: ground level (**a**) and tripod level (**b**).

As concerns the photogrammetric survey, it was carried out with the Ricoh Theta SC2 in about 4 h in two survey sessions; in particular, in the first session, the camera was positioned a few centimeters above the floor, while in the second session, about 1.5 m from the ground (Figure 6).

In this way, it was possible to obtain a twofold advantage, increasing the overlap of the images and capturing details that could not be acquired with a single low-level acquisition.

A total amount of 722 images was captured, of which 261 were taken at a height of about 20 centimeters above the floor from a small tripod, and 461 from a standard camera tripod.

**Figure 7:**
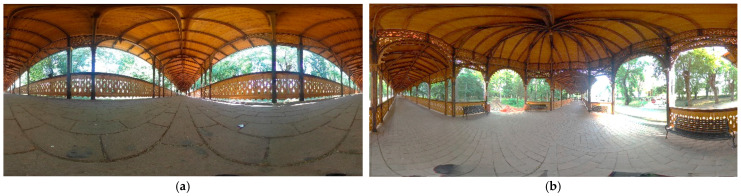
Examples of equirectangular images acquired by the Ricoh Theta SC2 camera from the ground (**a**) and from the tripod (**b**).

Some examples of the equirectangular image acquired by the Ricoh Theta SC2 on Buziaș Colonnade at ground level (Figure 7a) and on the tripod (Figure 7b) are shown in the following images.

**Figure 8:**
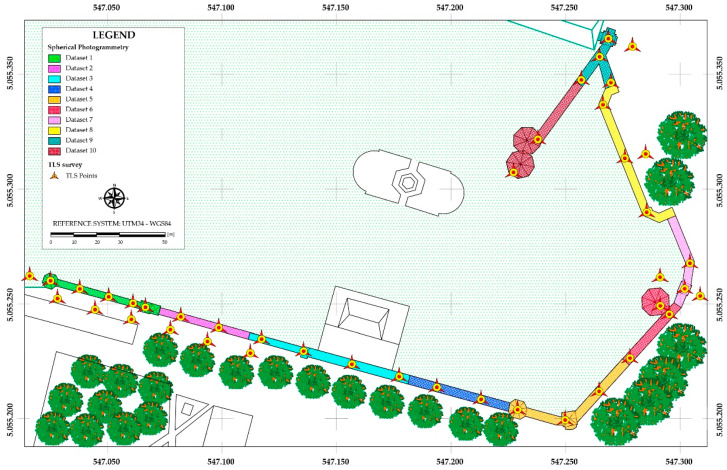
Plan with indication of photogrammetric datasets and TLS station positions.

The post-processing of the images was carried out, taking into account the result obtained in the laboratory test, in Agisoft Metashape software. Earlier to the image alignment step, a pre-processing of the images was carried out. In fact, in order to avoid effects on the images (chromatic aberrations, blooming) that could alter both the metric and radiometric quality of the images, appropriate masks were made. Furthermore, in order to improve the quality of the images, a pre-processing relative to the brightness, contrast, and exposure of the single images was carried out. Despite the high overlap of the images, the software was not able to align all the images at the same time. For this reason, it was decided to divide the acquired images spatially into various datasets. In particular, the complexity of the site and the quality of the sensor used for the experimentation made it necessary to process the data by discretizing the homogeneous geometric elements in order to obtain detailed photogrammetric models without having to incur problems related to the change of convergence of the morphology of the structure taken into consideration. The only way to elaborate a complete photogrammetric dataset of the site was to divide the images into 10 datasets. In this way, it was possible to obtain a 3D reconstruction of the site. From a statistical point of view, it was noted that only the division into 10 datasets (Figure 8) was able to give the best result. In other words, the dataset was first processed in a single process; subsequently, further, sub-datasets were created and processed individually until the best result was obtained through photogrammetric processing.

The parameters setting used for each dataset was: “Highest” (mean upscales the image by a factor of 4) for the Accuracy, the value of 40,000 as Key Point Limit (the maximum number of points the program will try to draw from an image) and the value of 4000 as Tie Point Limit (number of points the software will use to align photos to decrease processing time).

In addition, in order to cover areas in the images where the tripod or other elements outside the scene were visible, certain masks were made.

To georeference each dataset in the same TLS reference system, the coordinates of some points of the TLS point cloud easily recognizable on the images generated by the Ricoh Theta SC2 were extracted and used as Ground Control Points (GCPs) for the photogrammetric dataset.

The quality of the alignment phase of the different datasets was then evaluated as a function of the mean RMSE value which varied in a range between 15–20 cm; these RMSE values are partly due to the difficulty in identifying points (univocally) recognizable on the point cloud from the Ricoh Theta SC2 because, as discussed above, it was very noisy and not always able to describe in a geometrically correct way the objects detected.

Once the images were aligned, it was possible to build the dense point cloud for each dataset. To build the dense point cloud, the software uses several photo resolution scaling: Ultra High, High, Medium, Low, and Lowest. In other words, the build dense cloud settings are referred to as “Quality” in Metashape and impact the image resolution, where Ultra High processes the original images and subsequent settings downscale the images by increasing factors of 4; High downscales images by a factor of 4 (2× on each side), Medium by a factor of 16 (4× on each side), Low by a factor of 64 (8× on each side) and Lowest by a factor of 256 (16× on each side) [30].

**Figure 9:**
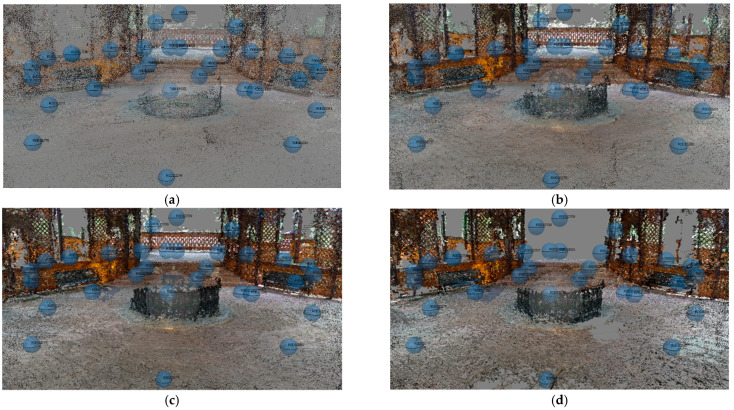
Dense Point Cloud of part of Buziaș dataset generated by Low (**a**), Medium (**b**) High (**c**) and Highest setting (**d**).

In order to choose the most suitable one for the dataset, several tests were carried out, varying the photo resolution scaling (Figure 9).

As shown in Figure 9, it can be seen that as the resolution of the images increases, the point cloud becomes denser and denser; however, as the point cloud is quite noisy, the high setting was used to build the point cloud of the various datasets.

## 4. Results

Once the different datasets were geo-referenced, the point cloud of the structure under consideration was constructed. In particular, a dense point cloud of approximately 23 million points was obtained. In order to evaluate the accuracy of the model generated by spherical images and processed with Agisoft Metashape software, a comparison between the point cloud obtained from the survey with the Ricoh Theta SC2 camera and that obtained from scans with TLS, was carried out. In particular, the evaluation was performed on a dataset, using the C2C algorithm implemented in Cloud Compare software. C2C is a method that performs a computation of distances between two clouds. The basic C2C distance calculation method calculates the nearest neighbor distance between the reference cloud and the datasets of the compared clouds. The nearest neighbor distance principle is used to calculate distances between two points where for each point in the compared cloud, the nearest point in the reference cloud is searched for and their Euclidean distance is calculated [32]. 

**Figure 10:**
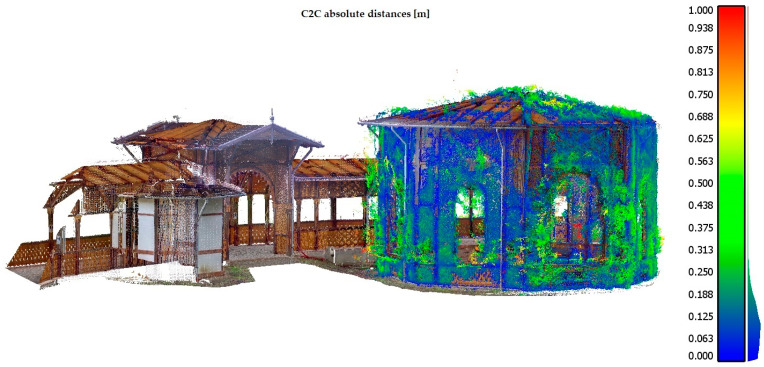
Evaluating accuracy between point clouds using the C2C algorithm: colored point cloud represents the one generated by TLS and is considered as a reference in the comparison while the point cloud in a chromatic scale that varies from green to red is generated by the photogrammetric process.

As shown in the following image (Figure 10), the maximum error value in the calculation of absolute distances is contained within 26 centimeters. This value obviously increases in correspondence with the areas in which the point cloud has more noise elements or outliers (points represented in Figure 10 in a chromatic scale that varies from green to red). In addition, 80% of the points were about 0.17 m away from the point cloud generated by TLS. Considering a Gauss distribution, it was possible to obtain a mean of 0.116 m and a standard deviation of 0.084 m.

## 5. Discussion and Conclusions

Spherical photogrammetry made it possible to build the 3D model of a structure (a long corridor) belonging to the CH rather quickly compared to TLS. In addition, process automation in SfM-MVS software is very fast and, as consequence, it was possible to build a 3D model with a good level of detail in a very short time.

The use of low-cost spherical cameras experimented with and described in the paper for 3D reconstruction through photogrammetric processes has not yet proved to be sufficiently suitable and efficient in obtaining a metrically accurate model. In fact, the model obtained is still affected by many noise disturbances and outliers, thus making the final result not appropriate for a highly detailed representation: in fact, in the survey of the site of Buziaș, it was necessary to acquire a large number of images to reconstruct the three-dimensional model of the site without, however, having a high degree of detail on particular finishes and shapes. The major problem that causes such a large error is the stitching algorithm to create a spherical image that does not fit the photogrammetric collinearity condition. It is particularly, the reference point in the middle of the portion of the image has better accuracy than reference points well distributed at the full frame of the image. The research of image stitching algorithms and RAW image management tools continues to be a constantly evolving field of research.

Certainly, one of the advantages offered by this type of sensor is the fast image acquisition phase and the amount of detail captured with a single shot. In addition, the ability to observe an entire scene around the camera in a single shot is a valuable tool for documenting a site under investigation and thus has the support of extra details during the restitution phases.

In order to reconstruct a 3D model using two spherical images, it was first necessary to develop an algorithm, as shown in Appendix A and subsequently, in the laboratory environment, a photogrammetric dataset was built and processed in SfM-MVS software; however, it was not possible to build, using this approach, a 3D model with sufficient detail. Therefore, spherical photogrammetry was used for the analysis and processing of the different sites taken into consideration.

In addition, from a color point of view, the camera was able to produce images with rather homogeneous contrast brightness; as a result, the point cloud also took on a pleasingly colorful appearance; the development of higher performance sensors undoubtedly increases the quality of the model and this becomes important when the details to be investigated are rather small and detailed complex.

From the experience conducted on the Buziaș site, it was noted that by using TLS, the reconstruction of the wood carvings of the Colonnade, and of all the geometrically complex objects present, was well defined and therefore very detailed. On the contrary, the detail of the carvings was not accurately reconstructed in the photogrammetric reconstruction phase, creating a lot of noise especially in correspondence of the holes in which the scene was represented in the background. This problem has therefore led to a point cloud that, although dense and acceptable from a colorimetric point of view, was not very detailed; in other words, this problem involved a non-faithful reconstruction of the characteristic geometric elements of the structure investigated. This means that, although very expensive, TLS surveying has allowed high precision and accuracy in the detection of details, particularly appropriate in the reconstruction of elements with complex and articulated shapes.

Therefore, the use of these low-cost sensors certainly represents an innovation in the field of the survey of CH; with the support of higher performance sensors and calibration parameters of the sensors themselves, in the near future, it will be possible to acquire photographic datasets for photogrammetric processing and then obtain highly performant and dimensionally accurate three-dimensional models, representing a valuable tool in the field of representation of CH also through the implementation of Virtual Tour applications. Indeed, spherical images can be used not only for photogrammetric purposes but also for the documentation of environments. In particular, the construction of virtual tours through the use of spherical images allows a simulated visit of a place and receive in real-time a series of additional information that can better interpret what is being observed [33]. Therefore, observing an element of historical-architectural heritage and documenting it through the use of these technologies allows to provide the end-user with a precise flow of information that can be made available in different fields of application such as tourism, and in different sectors such as design and research, including new communication environments such as those related to the World Wide Web [34,35]. This new "vision" related to documentation and information gathering was applied in the case study thanks to the availability of a 360° camera that allowed the creation of a virtual tour of the wooden colonnade of Buziaș. The photogrammetric process was used to generate the external orientation of the camera; in this way, it was possible to position the images along the route with high accuracy.

Finally, it is hoped that algorithms will be developed to improve the matching of spherical camera-generated images and, at the same time, tools and open-source software [36] will be implemented that can handle numerous spherical images and build detailed and accurate point clouds.

## Figures and Tables

**Table 1:** Features of typical commercial 360° cameras.

Product	Resolution [Megapixel]	Cost [€]	Level
Ssstar	16	200	entry
GoXtreme Dome 360	8	230	
Nikon KeyMission 360	23.9	280	
Xiaomi Mijia Mi Sphere 360	23.9	290	
GoPro Max	16.6	550	medium
Ricoh Theta V	14.4	550	
Garmin VIRB 360	15	700	
Vuze+	14.7	990	
Ricoh Theta Z1	22	1100	
Insta360 Pro 2	59	4000	high
iSTAR Fusion 360	50	5900	
Insta360 Titan	55	17,000	
Weiss Ag Civetta WAM2	230	not available	

**Table 2:** Technical features of Ricoh Theta SC2.

Specification	Description
Release date	12/2019
Exterior/external dimensions and weight	45.2 mm (W) × 130.6 mm (H) × 22.9 mm, 104 g
Still image resolution	5376 × 2688
Internal memory/Number of photos that can be recorded	Approx. 14GB
Object distance	Approx. 10 cm–∞ (from front of lens)
Exposure compensation	Still image: Manual compensation (−2.0–+2.0 EV, 1/3 EV step)
ISO sensitivity	Still image: (Automatic) ISO64–1600 (ISO priority mode) ISO64–3200 (Manual mode) ISO64–3200
Shutter speed	Still image: (Automatic) 1/25,000–1/8 s, (Shutter priority mode) 1/25,000–1/8 s (Manual mode) 1/25,000–60 s
Compression method	Still image: JPEG (Exif Ver2.3)
Lens configuration	7 elements in 6 groups
Lens_F value	value F2.0
Image sensor_size	1/2.3 CMOS (×2)
Effective pixels	12 megapixels (×2)
Output pixels	Approx. 14 megapixels

**Table 3:** Point coordinates (local system) obtained from TLS and estimation of photogrammetric errors.

Markers	X (m)	Y (m)	Z (m)	Error (m)	Error (pix)
1	1.879	−0.541	0.382	0.010	1.901
2	1.197	−1.388	2.523	0.029	1.749
3	1.611	−2.144	−0.091	0.027	1.752
4	0.319	−2.341	0.538	0.021	3.179
5	−0.569	−2.185	2.116	0.060	4.647
6	−1.300	−2.063	−0.007	0,022	1.826
7	−1.954	−1.953	1.314	0.032	1.765
8	−1.954	−1.042	2.509	0.033	2.328
9	−2.352	−0.686	0.043	0.022	2.014
10	−2.260	−0.100	2.132	0.015	2.096
11	−1.596	0.775	1.449	0.012	2.323
12	−1.064	0.729	2.503	0.033	2.905
13	−1.289	2.879	0.332	0.041	1.553
14	−0.404	2.168	1.380	0.033	3.161
15	0.100	2.110	−0.229	0.019	2.742
16	0.692	2.541	0.551	0.043	3.171
17	1.286	1.997	−0.074	0.029	1.287
18	1.848	1.869	1.348	0.038	2.216
19	1.960	2.341	2.184	0.042	0.662
20	0.939	1.237	2.525	0.036	4.149

**Table 4:** Comparison values between the two software versions.

Features	Agisoft Photoscan	Agisoft Metashape
Tie Points	1265	702
Dense Cloud	3,897,181	1,347,738
Error (m)	0.0320	0.0331
Error (pix)	2.569	3.210
Time alignment and match processing (s)	70 s	52 s

**Table 5:** Spatial configuration of spherical images and calculation of the increase in the number of tie points and dense clouds.

Dataset	Configuration	Tie Points	Increase of Tie Points	Dense Cloud	Increase of Dense Cloud
A	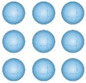 9 images at ground level	702	-	1,347,738	-
B	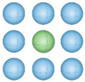 9 images at ground level + 1 image at tripod level	726	4%	1,552,328	15%
C	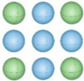 9 images at ground level + 4 images at tripod level	811	16%	2,091,827	55%
D	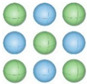 9 images at ground level + 5 images at tripod level	905	29%	2,246,343	67%
E	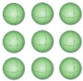 9 images at ground level + 9 images at tripod level	1164	66%	2,382,485	77%

## Appendix A

**Algorithm A1—Equirectangular to fisheye**
imequ2fish(path): img = readimg(path) emi_1 = img.crop((img.width/4, 0, 3 * img.width/4, img.height)) emi_2_1 = img.crop((0, 0, img.width/4, img.height)) emi_2_2 = img.crop((3 * img.width/4, 0, img.width, img.height)) emi_2 = emi_2_1 + emi_2_2 fish_1 = equ2fish(emi_1) fish_2 = equ2fish(emi_2) fish_1.save() fish_2.save() Where equ2fish is defined as: equ2fish(img): C = (img.with/2, img.height/2) foreach pixel in img: point = ((pixel.y – C.x)/C.x, (C.y − fishPos.x)/C.y) R = sqrt(point.x * point.x + point.y * point.y); if R <= 1: phi = R * aperture/2 theta = atan2(point.y, point.x) P = (R * sin(phi) * cos(theta), R * cos(phi), R * sin(phi) * sin(theta)) lon = atan2(P.y, P.x) lat = atan2(P.z, sqrt(P.x * P.x + P.y * P.y)) origPos = (lon/PI, 2 * lat/PI) origPixel = (C.x + origPos.x * C.x, C.y – origPos.y * C.y) if origPixel.x < img.height and origPixl.y < img.width: pixel = img(origPixel.x, origPixel.y)
